# Teacher Mindsets Concerning the Malleability of Intelligence and the Appraisal of Achievement in the Context of Feedback

**DOI:** 10.3389/fpsyg.2017.01594

**Published:** 2017-09-21

**Authors:** Emmy De Kraker-Pauw, Floryt Van Wesel, Lydia Krabbendam, Nienke Van Atteveldt

**Affiliations:** ^1^Behavioural and Movement Sciences, Vrije Universiteit Amsterdam Amsterdam, Netherlands; ^2^Methodology and Statistics, Utrecht University Utrecht, Netherlands; ^3^LEARN! Interfaculty Research Institute, Vrije Universiteit Amsterdam Amsterdam, Netherlands

**Keywords:** teacher beliefs, malleability of intelligence, mindset, appraisal of achievement, feedback

## Abstract

The pedagogical beliefs (e.g., beliefs or “mindsets” concerning the malleability of intelligence) that teachers hold may have a far-reaching impact on their teaching behavior. In general, two basic mindsets can be distinguished with regard to the malleability of intelligence: fixed (entity) and growth (incremental). In this article, we present two studies investigating the associations between teachers' mindset and (1) their appraisal of students' achievements and (2) the feedback they provide. Study 1 focuses on the associations between mindset and appraisal. The findings reveal an association between growth mindset and the appraisal of increasing student achievements. Study 2 investigates the impact of teachers' mindset on the amount and type of oral feedback they provide to their students. Contrarily to expectations, the findings reveal a significant negative correlation between mindset and the amount of feedback.

## Introduction

According to an increasing number of studies, the beliefs that teachers hold influence both their pedagogical decisions and their classroom behavior (Stipek et al., 2001; Cross, 2009; Jordan et al., 2010). Studies have addressed such aspects as the expectations that teachers have of students, their ratings of written and oral achievements and the feedback that they provide to students (Li, 1999; Andersson, 2012). Pedagogical beliefs can vary greatly, even amongst teachers within the same educational setting or school. One area in which differences in pedagogical beliefs could potentially have far-reaching effects has to do with the malleability of human attributes (e.g., intelligence; Howard-Jones, 2014). Such beliefs have also been referred to as mindsets concerning malleability (Dweck, 1999). The primary aim of our study is to investigate the association between teachers' mindset and their appraisal of students' achievements. The second aim is to investigate the association between mindset and the feedback teachers provide during real classroom lessons. Additionally, the role of the teacher characteristics gender and teaching domain are explored within these two main aims.

### Mindsets: is intelligence fixed or malleable?

In general, two basic mindsets can be distinguished with regard to the malleability of intelligence: the entity (fixed) mindset and the incremental (growth) mindset (Dweck, 1999, 2006). Some people believe that intelligence is a fixed trait: a person has a certain amount of intelligence, and it cannot be changed. The fixed mindset has been associated with performance goals and with helplessness-oriented strategies in response to failure and setbacks (Blackwell et al., 2007; Burnette et al., 2013), as errors are seen as confirming an individual's inability. Others believe that intelligence is malleable. In other words, intelligence can be changed through effort and persistence. This growth mindset is associated with learning goals, mastery-oriented strategies and beliefs in positive effort (Blackwell et al., 2007; Burnette et al., 2013).

To date, studies investigating the role of mindset in academic performance have focused largely on the mindsets of students with regard to their own intelligence. In this study, we address the mindsets of *teachers* concerning their general belief about the malleability of intelligence. The teachers' evaluation of students' achievements has consequences for the student's achievement motivation (Rheinberg, 1980, 1983, 2001). Teachers who compared students' learning outcomes with other students social reference norm (SRN), tended to attribute those academic achievements more often to stable characteristics of the student such as “ability.” Teachers who preferred intra-individual comparisons [the individual reference norm (IRN)] attributed academic achievements to highly variable causes such as “effort,” learning strategies and task characteristics. Dweck referred to these observations when she coined the terms fixed and growth mindset to describe the underlying beliefs people have about learning and intelligence (Dweck, 2006).

In the above mentioned study of Rheinberg (1980) on the influence of teacher's preferred reference norm on learning outcomes, he measured the following variables: students' achievement motive, IQ, and causal attribution of one's own academic success and failure at the beginning and at the end of the scholastic year. Additionally, at the end of the scholastic year, students rated the growth of their own academic competence during the year. Results showed that for teachers with an IRN low achievers at the beginning of the school year ended the year as moderate or even high achievers (Rheinberg, 1980). Contrarily, more than half of the students of teachers with SRN, who were low achievers at the beginning of the school year ended up as low achievers at the end of the school year.

Although it is important to note that definitions of the concept *intelligence* have been inconsistent and subject to change throughout the past century, the conceptualization of intelligence is not the subject of our current investigation.

### Appraisal of achievement and feedback

In this study, we hypothesize that the mindsets of teachers concerning the malleability of intelligence influences their appraisals of student achievement and the feedback they give. These two aspects of the classroom behavior of teachers (i.e., appraisal and feedback) might bear an influence on the learning outcomes and behavior of their students.

In this study, we understand “appraisal of achievement” as referring to the actual assessment of learning outcomes, which can be either preceded or followed by teacher feedback. Beliefs about ability and beliefs about effort are both important within the context of appraisal and feedback (Kaplan and Swart, 1973). *Feedback* can be defined in many different ways (Smith and Smith, 2015). In this study, we understand it as “information (provided by the teacher) on the performance of the learner, in which both the process and the result are important, in order to promote learning and maintain or increase motivation” (Brown, 2004). We discuss these concepts in greater detail in the introductions to the two studies.

### Teacher characteristics

Previous studies have suggested possible associations between the gender of teachers and their pedagogical behavior and beliefs (Nosek et al., 2002; Almutawa, 2005). For example, previous studies of teacher behavior have indicated that female teachers tend to stimulate collaboration and class discussion, in addition to being be more student-centered, indirect and supportive of students than is the case for their male colleagues (Li, 1999). Gender differences can also be observed in teacher feedback. A previous study has demonstrated that female teachers provide more supportive and more expressive feedback (in equal amounts to boys and girls), as compared to male teachers (Duffy et al., 2001). In another study, female teachers gave more compliments and used less directive forms of feedback than male teachers did (Rashidi and Naderi, 2012). Gender has further been shown to affect teachers' beliefs on, for example, the nature of particular school subjects, the curriculum and conceptions of the teaching role (Li, 1999). Less is known, however, about the effects of teacher gender on beliefs concerning the malleability of intelligence, and the appraisal of achievement and feedback.

A second teacher characteristic, *teaching domain*, is likely to be related to mindset, given the tendency of people to assume that success in some domains (e.g., science, technology, engineering, and mathematics—STEM—subjects) depends upon innate ability, even more so than on dedication and perseverance. Women tend to be stereotyped as lacking such ability (Leslie et al., 2015). Characteristic “teaching domain” had to be taken into account for several reasons, such as the gender-specific character of some school subject (Vassilou, 2010), low proportions of women entering the STEM domain (Meelissen and Drent, 2009; Michels et al., 2014) and the presence of strong gender science stereotypes in men dominated science fields specifically (Leslie et al., 2015). Additional reasons to take domain into account are the more reported negativity in interactions between STEM teachers and their students (Watt et al., 2013) and the negative motivation of STEM teachers to choose teaching as a fallback career (fallback career: second choice career, when one has “failed to be accepted into the career of choice or otherwise unable to pursue their first-choice career,” Watt et al., 2013).

### Overview of the article

The primary aim of the article is to investigate how mindset influences (1) the appraisal of student achievement (study 1) and (2) the frequency and type of oral feedback that teachers provide in the classroom (study 2). Additionally, the effects of gender and teaching domain on appraisal of achievement and feedback are explored.

## Study 1

### Background: appraisal of achievement

The ways in which teachers evaluate and appraise the achievements of their students might depend upon the reference norms (e.g., individual vs. social comparisons) they prefer to apply when evaluating learning outcomes. As established by Rheinberg (1980), the concept of *teacher reference norm orientation* (RNO) is defined as “a standard to which individual achievements are compared.” Such standards can be based on any of the various frames of reference that teachers can adopt. The reference norm can be seen as an effect of a teacher's mindset concerning malleability. According to Heckhausen (1974) three frames of reference have been distinguished: the SRN, the IRN and the criterial reference norm (CRN). When applying the SRN, teachers compare the achievements of a given student to those of fellow students. Teachers adopting the SRN believe that differences in ability amongst students are highly stable across time. Their appraisals are strongly dependent upon whether the learning outcomes of a given student are above or below the class average (Rheinberg and Engeser, 2010).

When applying the IRN, teachers compare the achievements of individual students to their prior achievements. Teachers adopting the IRN emphasize improvement, effort and learning, with a focus on the individual process of learning. The application of this reference norm has been shown to decrease fear of failure in students and brought many low achievers up into the high achieving range (Rheinberg and Engeser, 2010). When applying the third type of reference norm, the CRN, task-inherent properties are taken as the standard of comparison. In a study of Martinez et al. (2009) it was found that teachers evaluated student performances not in absolute terms but relative to other students in the school (SRN) and that they might adjust their grading for some students, perhaps with basis on perceived differences in needs and/or abilities.

Based on a recent study of teachers working with students with learning disabilities and otherwise academically-challenged students, Wilbert and Grúnke (2010) report that the reference norms used by teachers can affect the achievements and motivation of their students, with the INR having a positive effect on learning and the SNR inhibiting learning.

### Specific aim and hypotheses

The current study addresses a gap in the research literature with regard to the relation between mindsets of teachers concerning the malleability of intelligence, teacher characteristics gender and teaching domain, and their appraisal of achievement. The IRN as mentioned above is growth-oriented. Teachers with an IRN appraise improvement, effort and learning, similar to growth-oriented teachers. We expect growth- oriented teachers to value increasing achievements positively, independent from the end marks students achieve. This in contrast to fixed oriented teachers; we expect them to value sufficient end marks, and we expect them to have less appraisal for students' personal increasing improvements. Therefore, we tested the main hypothesis that teachers with a more growth- oriented mindset are more positive in their appraisal of students' increasing achievements than those with a more fixed oriented mindset.

In addition, the effects of teacher characteristics “gender” and “teaching domain” on their appraisal of achievement were explored.

### Method

#### Participants

The study was presented in a meeting for managers from 11 secondary schools in the southwest of the Netherlands. Ten of these schools expressed their willingness to participate in the study. The manager of each school received a letter containing an explanation of the study, along with a description of its aims and a global timeline. A presentation was given to the delegates of the school teams. In addition, all participants received a letter containing information on the study. To guarantee that participation of teacher participants was always on a fully informed and voluntary basis, we obtained active informed consent from them prior to onset of the studies. Dutch legislation lays down procedures for the ethical review of medical research involving human subjects. However, this study does not fall under the current definition of medical research and therefore formal approval by an ethics committee is not legally required. The ethics committee of the Faculty of Behavioral and Movement Sciences at Vrije Universiteit Amsterdam has issued guidelines to foster the adherence to ethical principles for the non-medical behavioral research within the faculty, and the current study has been conducted in strict accordance with these guidelines.

In all, 106 teachers participated in the study. The participating teachers worked primarily with second-year students (13–15 years of age) in schools for preparatory secondary vocational education.

We investigated the association between mindset on malleability of intelligence and appraisal of achievement and the two factors 1) gender: male (*n* = 63) vs. female (*n* = 43), and 2) teaching domain: STEM (*n* = 27) vs. non-STEM (*n* = 79). The teachers ranged in age from 22 to 61 years (*M* = 42.03, *SD* = 11.76), with teaching experience varying between 1 and 40 years. Of all participating teachers, 65% were working in public schools, while 34% were working in religious (Christian) schools. For 1% of the participants, the type of school was unknown.

#### Instruments

Each participant completed two online questionnaires, which they accessed through an anonymous survey link. The first instrument was the Theory of Intelligence Questionnaire (TOI), which is designed to measure implicit beliefs about the malleability of intelligence. The questionnaire consists of three “entity theory” statements and three “incremental theory” statements (Dweck, 2007).

All items were scored along a Likert-6 scale ranging from “completely agree” to “completely disagree.” The incremental items were reverse-scored, such that low scores represent a “fixed” mindset and high scores represent a “growth” mindset on all six items. The internal consistency of this questionnaire for the current sample was high, with a Cronbach's alpha value of 0.90.

**Figure 1:**
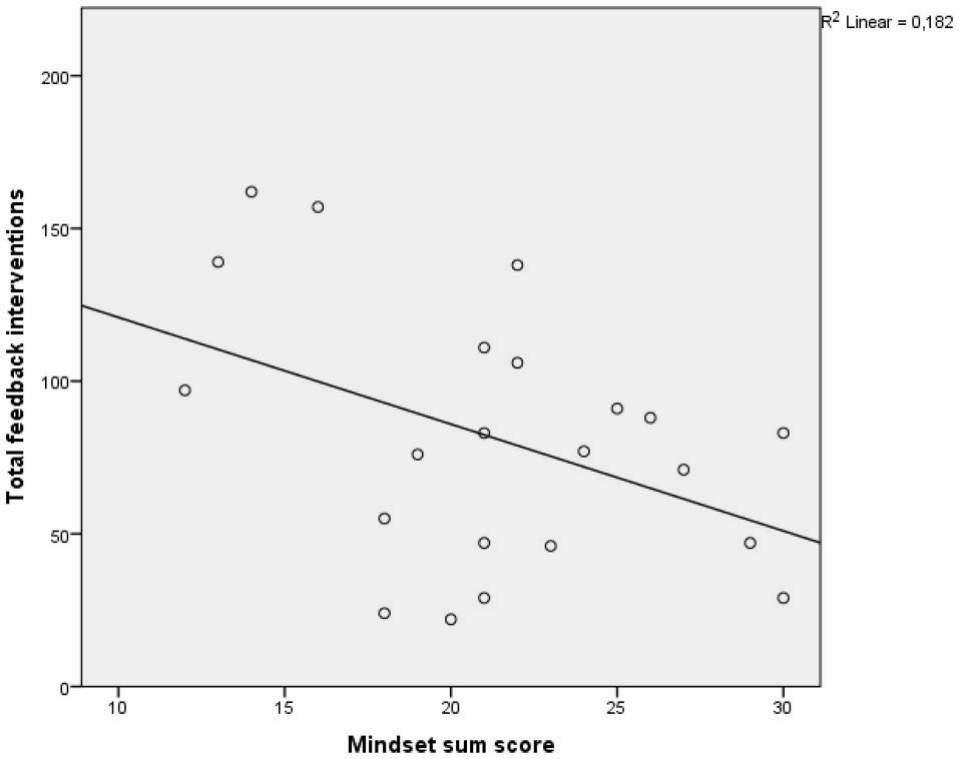
Scatterplot correlation between total feedback interventions and mindset score.

The second questionnaire was the Rheinberg's Reference Norm Orientation Test (Rheinberg, 1980). In its original version, three sequential test results from nine fictional students are presented. The students' test scores in the original Reference Norm Orientation Test were either increasing, stable of decreasing. The teacher's task is to evaluate the result each student had in the third test. If he evaluates a result as “good achievement,” he can mark between one and five plus signs into the five boxes coordinated on the right side. If the teacher evaluates a result as “poor achievement,” he can mark between one and five minus signs into the five boxes. No signs means a neutral evaluation. The way the teachers evaluate the final test score reflects to what extent they emphasize individual improvement (IRN) vs. a comparison to fellow students (SRN). We made several adaptations to the test, to make it better suited to our goal of investigating the appraisal of increasing achievement (even if the last test score was an insufficient mark). A positive appraisal of increasing achievement is predicted to be associated with a growth mindset. Our adaptations included the elimination of one set of scores because it resembled another set and the addition of four new sets of scores with larger intervals between the three marks in order to generate more pronounced increasing or non-increasing scores (see Appendix 1, Figure 1).

The instrument used in the current study thus distinguished three types of individual achievement: (a) increasing marks, (b) non-increasing, insufficient mark (<5.5), and c) non-increasing, sufficient mark (≥5.5). Participants could rate the last test score on a scale (− −. −, ±, +, ++) from − − (poor achievement) to ++ (good achievement) with ± indicating a neutral evaluation. Reliability analysis in our sample resulted in a Cronbach's alpha value of 0.81 for the increasing marks, a value of 0.76 for non-increasing sufficient marks and 0.66 for non-increasing insufficient marks.

#### Data analysis

We used the Statistical Package for the Social Sciences (SPSS) version 20.0 for Windows in all data analyses. We calculated the score on the Theory of Intelligence Questionnaire (TOI) for each individual by taking the sum of the scores on all six items (incremental items were reverse-scored). The Reference Norm Orientation (RNO) scores were calculated by recoding the appraisal of achievement scores for all items (−− = 1; − = 2; ± = 3; + = 4; ++ = 5). We then calculated mean scores for the three subscales: (a) increasing marks, (b) non-increasing sufficient mark, and (c) non-increasing insufficient mark.

To examine the relationship between mindset and the appraisal of achievement, we first present descriptive statistics. Furthermore, we present the bivariate correlations between the mindset sum score and each subscale of the appraisal of achievement test, as well as it's relation with gender and teaching domain. Second, we investigated our main hypothesis by using multiple linear regressions to test the effect of mindset (sum scores) on the increasing scale of appraisal of achievement, while also taking gender and teaching domain into account.

### Results

#### Descriptive statistics including bivariate relations

In Table 1, the means and standard deviations of the three RNO subscales are presented for male (*n* = 63), female (*n* = 43), STEM (*n* = 27) and non-STEM (*n* = 79) participants and the total scores for increasing (*M* = 3.71, *SD* = 0.60), non-increasing sufficient (*M* = 3.53, *SD* = 0.54) and non-increasing insufficient marks (*M* = 2.20, *SD* = 0.45).

**Table 1:** Means and standard deviation for Mindset (*n* = 115), Appraisal of Achievement (*n* = 106), broken down for male, female, STEM and non-STEM participants.

	**Theory of intelligence**					**Appraisal of student achievement**												
							**Increasing**				**Non-increasing sufficient**				**Non-increasing insufficient**			
	**n**	**Min**.	**Max**.	**M**	***SD***	**n**	**Min**.	**Max**.	**M**	***SD***	**Min**.	**Max**.	**M**	***SD***	**Min**.	**Max**.	**M**	***SD***
Male	65	11	36	19.52	6.02	63	2.25	4.75	3.54^*^	0.60	2.00	4.75	3.56	0.57	1.50	3.00	2.15	0.47
Female	50	12	30	21.76	4.70	43	2.25	4.75	3.95^*^	0.51	2.50	4.75	3.48	0.51	1.50	3.25	2.27	0.41
STEM	28	12	36	20.14	6.08	27	2.25	4.75	3.73	0.58	2.25	4.75	3.53	0.55	1.50	3.00	2.25	0.46
Non-STEM	87	11	36	20.61	5.43	79	2.25	4.50	3.70	0.60	2.00	4.75	3.53	0.54	1.50	3.25	2.18	0.45
Total	115	11	36	20.50	5.57	106	2.25	4.75	3.71	0.60	2.00	4.75	3.53	0.54	1.50	3.25	2.20	0.45
Mindset						106			*r* = 0.24 *p* = 0.01				*r* = −0.06 *p* = 0.52				*r* = 0.09 *p* = 0.36	

*The bottom row indicates the correlation with mindset sum scores*.

* *p < 0.05. Significance of t-test comparing gender or domain*.

We calculated a Pearson product-moment correlation coefficient to determine the association between mindset (*M* = 20.50, *SD* = 5.57) and the appraisal of achievement for the increasing, non-increasing sufficient and non-increasing insufficient marks (see final row of Table 1). For increasing marks (*r* = 0.24, *n* = 106, *p* = 0.013) there was a significant, albeit weak, positive correlation between the two variables. This positive correlation indicated that higher scores on mindset (more growth-oriented) were associated with a higher appraisal of increasing marks. No significant correlations were found between mindset and the appraisal of achievement for the non-increasing sufficient marks (*r* = −0.06, *n* = 106, *p* = 0.52) or the non-increasing insufficient marks (*r* = 0.09, *n* = 106, *p* = 0.36*)*.

Additionally, Table 1 shows results of *t*-tests comparing RNO scores (appraisal) between male and female teachers and STEM and non-STEM teachers. As can be seen in this table, female teachers showed higher scores on increasing marks (*M* = 3.95, *SD* = 0.51) than male teachers did (*M* = 3.54, *SD* = 0.60). This difference was significant (*t* = −3.59, *df* = 104, *p* < 0.001). Differences between female and male teachers on non-increasing sufficient and non-increasing insufficient marks were not significant (*t* = 0.77, *df* = 104, *p* = 0.44 and *t* = −1.37, *df* = 104, *p* = 0.18 respectively).

Results showed no significant differences on increasing marks between STEM and non-STEM teachers (*t* = −0.24, *df* = 104, *p* = 0.81), no significant differences on non-increasing sufficient marks (*t* = −0.02, *df* = 104, *p* = 0.98*)*, nor on the non-increasing insufficient marks (*t* = −0.73, *df* = 104, *p* = 0.47).

#### Hypothesis testing: the effect of mindset, gender and domain on the appraisal of achievement

In order to test the main hypothesis, the effect of mindset (sum scores) on the increasing scale of appraisal of achievement, while also taking gender and teaching domain into account, we conducted a multiple linear regression analysis. This analysis showed that gender (β = 0.32, *t* = 3.396, *p* < 0.001) and mindset (β = 0.19, *t* = 2.034, *p* = 0.05) were significant predictors, with female teachers and teachers with a more growth-oriented mindset giving higher appreciations. The association with domain was non-significant (β = 0.10, *t* = 1.033, *p* = 0.30).

### Discussion

In this study, we investigate the association between the mindsets of teachers and their appraisal of student achievements. In line with our hypothesis, the results reveal a positive correlation between mindset and the appraisal of achievement for the increasing marks but not for non-increasing marks. Attention for increasing student achievements has been demonstrated to be of importance for students' motivation (Meece et al., 2006; Rheinberg and Engeser, 2010; Wilbert and Grúnke, 2010). In addition, we found that gender is associated with the appraisal of increasing marks, with women valuing these achievements slightly higher than men. In the following section, we present Study 2, exploring how mindset is related to the type and amount of feedback that teachers provide to their students in daily classroom situations.

## Study 2

Study 2 focuses on the oral feedback provided by teachers in classroom interactions, with the goal of identifying possible associations between the general mindset that teachers have concerning the malleability of intelligence and the feedback interventions that they use.

### Feedback: growth and fixed

In classroom situations, teachers generally *provide* feedback, to which students *respond*. The feedback that teachers provide to their students affects their learning behavior and learning outcomes (Hattie and Timperley, 2007; Lipnevich and Smith, 2009; Geyskens et al., 2012), and it has a powerful influence on motivation (Hattie and Timperley, 2007; Wilbert et al., 2010).

The literature contains a variety of classifications regarding types and descriptions of feedback. We highlight several of these classifications in the context of our study. One classification has to do with the forms, objects, descriptions, opinions, views, effects and goals of feedback (Sol and Stokking, 2009). In their scoring form for the Observation of Teacher Feedback Behavior, Sol and Stokking (2009) list result-oriented, process-oriented, instruction-oriented and “other” feedback. Emphasizing that it is important for students to *understand* the information they receive, Hattie and Timperley (2007) distinguish four levels of feedback: task, process, self-regulation and self-level.

Beliefs in general influence behavior (Cross, 2009). Earlier research shows that feedback both regulates and is regulated by motivational beliefs. External feedback has been shown to influence how students feel about themselves (positively or negatively), and what and how they learn (Dweck, 1999).

To monitor the progress of and the reflection on the students' learning process, teachers should provide formative feedback. This type of feedback provides information on performance to improve and accelerate learning (Sadler, 1998) and to adjust teachers' educational activities (Sluijsmans et al., 2013). The research on formative assessment and feedback was reinterpreted by Nicol and Macfarlane-Dick (2006) to show how these processes could help students take control of their own learning, i.e., become self-regulated learners. In this way, feedback not only contributes to the teaching process, but also to the learning process and the improvement of achievements (Arts et al., 2016; Schildkamp et al., 2016). In some countries high-ranking on PISA scores (e.g., Finland), self-regulating skills and the continuous providence of formative feedback to students is central to the learning process (Hill, 2011).

Feedback is closely related to assessment: earlier studies showed that tests focused on certification and selection, without forms of informative feedback were negative for the process of learning. Without informative feedback those tests diminished student's responsibility and motivation for learning (Sluijsmans et al., 2013).

Corresponding to the description of a growth mindset, growth-oriented feedback has been described as feedback that guides and motivates students, enhances their learning (Voerman, 2014), and keeps them persistent, resilient and focused on the process of learning. It provides specific information (Voerman, 2014) about the progress (and results) of students. Corresponding to the description of a fixed mindset, fixed feedback emphasizes basic qualities (e.g., intelligence or talent) and characteristics as fixed traits (Kamins and Dweck, 1999). It provides information about the results as such, and not about the process of learning.

In conclusion, and consistent with the conclusions of Hattie and Timperley (2007) and of Shute (2008), we regard feedback as information provided by the teacher concerning the performance of the learner, with both process and result being important, in order to promote learning and to maintain or increase motivation (Brown, 2004).

Based on the literature above, we distinguish between two dimensions of oral feedback: growth-oriented feedback and fixed feedback.

Growth-oriented feedback:

- Personal praise and criticism for *doing* (“well done, you tried very hard”), for efforts made or strategies chosen.
- Process-oriented: Comments on *how* results have been achieved and can be improved.
- questions regarding strategies, efforts, possible improvements, alternatives for choices (Kamins and Dweck, 2012), hints, cues, dividing in small steps, prompts, suggestions for improvement and monitoring the process (Sol and Stokking, 2009).

Fixed feedback:

- Personal praise and criticism for *being* smart, quick, stupid (“you are a very intelligent person”), feedback directed to traits, characteristics or abilities.
- Results-oriented: Comments on *what* results have been achieved: correct or wrong answers, giving the correct answer and indicating *what* is missing.

### Specific aims and hypotheses

Study 2 focuses on the oral feedback provided by teachers in relation to their mindsets. First we investigated which types of feedback interventions teachers generally provide in their classrooms (based on the Observation of Teacher Feedback Behavior instrument (Sol and Stokking, 2008) and our definition of growth and fixed feedback as described above. Then we hypothesized that teachers with a growth mindset would overall provide *more* feedback than teachers with a fixed mindset would (Hypothesis 2a), given that those with a growth mindset believe that feedback on efforts and strategies *during the learning process is essential* to the learning and achievement of students. For the same reason, we hypothesized that the growth- oriented teachers would provide more growth-oriented feedback (Hypothesis 2b) than fixed feedback.

Furthermore, we investigated the effects of two teacher characteristics, gender and teaching domain, with regard to the type and amount of feedback provided.

### Method

#### Participants

A subgroup of 23 teachers (12 male, 11 female) from the sample used in Study 1, all teachers teaching the second-years students (13–15 years old), in mathematics (*n* = 11) or Dutch (12), took part in classroom observations (video recorded). The length of their teaching experience varied from 1 to 40 years. The video recordings of one teacher could not be used, due to technical failures.

Comparison of this sample (*n* = 23) to the original sample (*n* = 106) revealed no significant differences with regard to mindset scores or the appraisal of achievement.

#### Procedure

All participants were informed about the video procedure in a personal conversation, during which the date and time of the video observations were agreed upon. Each school had a video protocol that required parental permission for any video recordings in classrooms. Next, parents were asked for permission to let their children participate in the research through an informed consent form. The teachers were able to identify the students whose parents had not given permission for the video observations, and these students were not included in the video recordings. The participants were encouraged to teach as “normally as possible,” and no special educational situations were created. Two video cameras were used: one was placed on a stand (permanent position), and one was used by the researcher to make close-up recordings of feedback moments. To guarantee that participation of teacher participants was always on a fully informed and voluntary basis, we obtained active informed consent from participants prior to onset of the studies (in strict accordance with the guidelines to ethical principles of Vrije Universiteit Amsterdam, see also Section Participants). Teacher participants received a letter with specific information about the research and ethical procedures such as the storage of the audio and video material (saved in a locked cabinet) and data, the confidentiality of personal information, and the report of results and conclusions afterwards.

#### Instruments

The video observations were conducted during mathematics or Dutch lessons in schools of preparatory secondary vocational education. The ages of the students ranged from 13 to 15 years. All lessons were taped in their entirety and transcribed verbatim. Some lessons started or ended with a brief conversation or announcement by the teacher. These interactions were not considered further, thus leading to variation in the length of the video fragments (see Appendix 2, Table 1).

The current study required an instrument that would allow us to code growth feedback and fixed feedback. Based on the literature above, we selected the scoring form for the Observation of Teacher Feedback Behavior (Appendix 2, Figure 1) developed by Sol and Stokking (2008) in order to score the feedback. This form distinguishes four categories of feedback for classifying and counting all feedback interventions: result-oriented (RO), process-oriented (PO), instruction-oriented (IO) and “other” (Cronbach's alpha = 0.77). This form came close to what we needed in order to score the growth feedback and fixed feedback provided by the participants, but two aspects were lacking. For this reason, we made two additions. First, two researchers independently assessed all comments expressing personal praise and criticism for *doing* (“well done, you tried very hard”) and *being* (“you are a very intelligent person”) from the transcriptions. The researchers then independently labeled each transcribed personal feedback comment as either growth or fixed praise/criticism. In 93.9% of these interventions, the two researchers assigned similar scores (Krippendorff's alpha = 0.92).

In a second step, we analyzed growth feedback reflecting the assessment of *how* results had been achieved. To this end, we used 8 of the 10 items from the category of *process-oriented* feedback from the original Teacher Feedback Behavior scoring form (Sol and Stokking, 2008), see Appendix 2, Figure 2. Two items from the original scoring form (“asking questions about knowledge” and “asking the question Do you understand?”) did not represent growth feedback. We therefore re-labeled these two items as “other process.”

For the fixed comments regarding *what* results had been achieved, we used all items of the category of “result-oriented” feedback from the original scoring form.

To establish inter-rate reliability in comments concerning “how” (growth) or “what” (fixed) results were achieved, two researchers scored the first 10 min of the video recordings of four teachers (selected at random), using the adapted version of the scoring form (see Appendix 2, Sol and Stokking, 2008; inter-rater reliability: Krippendorff's alpha = 0.88). Thereafter, one researcher scored 13 recordings, and the other scored 9.

#### Data analysis

To examine the relationship between mindset and the feedback, we first present descriptive statistics of types of feedback interventions teachers generally provide in their classrooms (based on the Observation of Teacher Feedback Behavior instrument, Sol and Stokking, 2008) and our definition of growth and fixed feedback as described above.

The variable “total feedback interventions” was created for each teacher by taking the sum of all feedback interventions provided by that teacher: feedback on instructions, behavior, fixed (personal praise/criticism and result interventions), growth (personal praise/criticism and process interventions) and other (questions about knowledge such as “do you understand”?) feedback (see Table 2). The variables “growth” and “fixed” feedback were created as follows: The growth feedback interventions include the personal praise/criticism for doing as well as the process growth interventions. The fixed feedback includes the personal praise/criticism for being as well as the fixed result interventions. Next, we calculated the *proportion* of fixed and growth feedback interventions as dependent variable in our analyses: the number of fixed or growth interventions teacher x/ total number of feedback interventions teacher x. This gives the relative amount of fixed or growth feedback interventions provided by a teacher as a proportion of the total amount of provided feedback by that same teacher, and thereby corrects for individual differences between teachers with regard to how much feedback they provide overall.

**Table 2:** Frequencies of oral feedback interventions, personal growth and fixed praise/criticism, growth-oriented with regard to “how,” fixed with regard to “what,” other process-oriented, and other behavior from 22 teachers (as observed on video).

**Feedback interventions**	**Frequency**	**Percentage of total feedback interventions**	**Number of teachers**
Personal growth praise/criticism on “*doing”*	23	1.26	12
Personal fixed praise/criticism on “*being”*	23	1.26	9
Growth-oriented feedback on *how* results were achieved	483	26.5	22
Fixed feedback on *what* results were achieved	503	27.6	22
Instruction-oriented	72	3.9	17
Other process-oriented Asking questions about knowledge Asking questions like, “Do you understand”?	279	15.0	19
Other behavior	442	24.2	22
Total	1,824	100	

Next, we address our hypotheses by reporting the bivariate correlations (Pearson's product-moment correlation coefficient) between mindset scores and the total number of feedback interventions (Hypothesis 2a) and between mindset scores and the two specific types of feedback (growth and fixed) (Hypothesis 2b). For testing hypothesis 2a we used the (sum of) the total feedback interventions as dependent variable.

For testing hypothesis 2b we used the *proportion* of fixed and growth feedback interventions as dependent variable in our analyses.

Furthermore, we investigate the effect of the teacher characteristics gender and domain on total amount of feedback and the proportions fixed and growth using independent sample *t*-tests.

### Results

#### Descriptive statistics

In all 22 video-taped lessons together (11 male teachers, 11 STEM-teachers, with mean mindset sum score *M* = 21.45) we identified a total of 1,824 oral feedback interventions. Table 2 presents the frequencies of the types of these oral feedback interventions from the 22 teachers (as observed on video).

The median of the total feedback interventions was 80.00. Total feedback interventions included feedback on instructions, behavior, fixed (personal praise/criticism and result interventions), growth (personal praise/criticism and process intervention) and “other process” (questions about knowledge such as “do you understand”?) feedback. 483 of all oral feedback interventions were categorized as growth feedback concerning *how* results had been achieved (remarks on strategies, efforts etc., for example “Can you tell me how you discovered the solution?”; “That's an interesting idea….let's try”; “You don't have to do it immediately right”; “What strategy can you use to ……?”; “Aaah, how did you find this answer?”; “What do you need first?”). All 223 teachers provided this type of feedback with a median 17.50 times per teacher. Only 23 of the total number of interventions could be categorized as growth-oriented personal praise (18)/criticism (5) for *doing* (persistence and effort). Twelve teachers provided this type of feedback. So, taken together the personal praise/criticism for doing and process-feedback, 27.8% of all feedback provided by the teachers was identified as growth feedback.

Of the 1,824 oral feedback interventions observed, 503 interventions could be categorized as fixed feedback regarding *what* results had been achieved (e.g., “That's just not good”; “No, wrong”; “Yes, the right answer is 70%”; Yes, ok, that word ends with a “d”). All 22 teachers provided this type of feedback with a median of 21.50. Of all oral feedback interventions, 21 were fixed personal praise interventions (for *being* smart, quick etc., for example: “student: “what if I am just smart?” teacher: yes, you are smart”), and 2 were fixed criticism interventions (for *being* slow, stupid etc.). 9 teachers provided this type of feedback. Taking together the personal fixed comments and the result oriented feedback, 28.8% of all feedback interventions were categorized as fixed feedback. All participants provided this type of feedback (see Table 2). 442 interventions were categorized as feedback on behavior, 72 on instruction. Table 3 presents means and standard deviations for the total number of feedback interventions, and for the proportions of fixed and growth feedback, including means and standard deviations for these proportions for gender and domain separately.

**Table 3:** Means and standard deviation, minimum and maximum for total feedback interventions, proportion growth feedback, proportion fixed feedback and broken down for male, female, STEM, non-STEM teachers.

		**Total feedback interventions**				**Proportion growth feedback**				**Proportion fixed feedback**			
	**n**	**Min**.	**Max**.	**M**	***SD***	**Min**.	**Max**.	**M**	***SD***	**Min**.	**Max**.	**M**	***SD***
Male	11	22	162	82.36	53.29	0.09	0.53	0.31^*^	0.14	0.05	0.73	0.34	0.18
Female	11	29	138	79.27	29.89	0.13	0.35	0.21^*^	0.07	0.17	0.66	0.36	0.15
STEM	11	29	162	89.55	40.07	0.15	0.53	0.32^*^	0.11	0.05	0.43	0.29	0.12
Non-STEM	11	22	157	72.09	44.33	0.09	0.46	0.21^*^	0.10	0.17	0.73	0.40	0.18
Total	22	22	162	80.82	42.19	0.09	0.53	0.26	0.12	0.05	0.73	0.35	0.16

* *p ≤ 0.05. Significance of t-test comparing gender or domain*.

#### Hypothesis testing (bivariate correlations and t-tests)

We calculated bivariate correlations (Pearson product-moment correlation coefficient) to determine the association between mindset and the total number of feedback interventions (Hypothesis 2a) and the two different types of feedback (proportion growth and proportion fixed) (Hypothesis 2b). A significant negative correlation was found between mindset score and the total feedback interventions (*r* = −0.43, *p* = 0.05*)* (see Figure 1) indicating that the more teachers' mindsets were growth-oriented, the less feedback they provided. With regard to the proportion of the two types of feedback (growth-oriented or fixed oriented) the analysis showed no significant correlations (*r* = −0.37, *p* = 0.09 and *r* = 0.24, *p* = 0.28 respectively). Additionally, Table 3 shows results of *t*-tests comparing the total number and the proportions for specific feedback types between male and female teachers and STEM and non-STEM teachers.

No significant differences were found between male and female teachers with regard to the total number of feedback interventions (*t* = 0.168, *df* = 20, *p* = 0.87) nor between STEM and non-STEM teachers (*t* = −0.969, *df* = 20, *p* = 0.34). However, male teachers provided a significantly higher proportion of growth feedback than female teachers did (*t* = 2.129, *df* = 20, *p* = 0.05). Furthermore, significant differences were found between STEM and non-STEM teachers with regard to the growth feedback (*t* = −2.304, *df* = 20, *p* = 0.03) indicating that STEM teachers provide a higher proportion of growth feedback then non-STEM teachers. Note that gender and domain are related in the current sample (*Chi*^*2*^ = 4.54, *df* = 1, *p* = 0.03) such that there are more male teachers in the STEM and more female teachers in the non-STEM domain.

### Discussion

The second study was designed to investigate the link between the mindsets of teachers and the amount and type of feedback they provided in classroom situations. Personal praise or criticism (growth and fixed) was used in only 2.5% of all oral feedback interactions. Feedback concerning *how* (growth) and *what* (fixed) was provided in almost equal amounts (26.5 and 27.6% respectively). Growth-oriented and fixed feedback together comprised roughly half of all of the feedback interventions observed (with the other half being related to instruction or behavior). Contrary to our hypothesis, we found a significant negative correlation between mindset score and the total amount of feedback. In other words, teachers with a more growth- oriented mindset provided less feedback than teachers with a more fixed oriented mindset. Furthermore, we found an indication that male and/or STEM-teachers provided more growth feedback compared to female/non-STEM teachers. In the general discussion below, we address our findings from Studies 1 and 2 in an integral manner.

## General discussion

The two studies presented aimed to investigate the relationship between the mindsets of teachers and their appraisal of student achievements (Study 1) and between mindset and the amount and type of feedback provided (Study 2). In addition to teachers' mindset, we also explored how specific teacher characteristics (i.e., gender, teaching domain) are associated with their appraisal of achievements and the feedback provided to students.

The relationship between beliefs and practices is complex and controversial (Savasci-Acikalin, 2009). The complexity of the relationship might be at least partly due to variety of belief definitions in literature (Bingimlas and Hanrahan, 2010), various pedagogical subjects or domain specificity. The findings have not been consistent (Fang, 1996; OECD, 2009; Bingimlas and Hanrahan, 2010; Saad and Boujaoude, 2012; Mansour, 2013). For example, regarding beliefs and practices in mathematics, some researchers reported consistencies (Stipek et al., 2001; Kuzborska, 2011; Ertmer et al., 2012; Zakaria and Maat, 2012; Polly et al., 2013) whereas others reported inconsistencies between teachers' beliefs and educational practices (Beswick, 2004; Kynigos and Argyris, 2004; Li and Yu, 2010).

To the best of our knowledge there are no results of previous research regarding the association between teachers' beliefs about malleability of intelligence (growth or fixed mindset) and the feedback behavior they provide to their students. The current study investigates the associations between beliefs and behavior through an appraisal of achievement test and classroom observations to fill existing gaps in the literature: First, beliefs about the nature of intelligence might have far-reaching effects (Howard-Jones, 2014). Second, direct observation of lessons and of teachers' decision-making and goals may be instrumental to understand their beliefs (Bingimlas and Hanrahan, 2010). Third, teachers' self-evaluations on teaching practices, might not reflect actual classroom practices (Ertmer et al., 2012).

### Appraisal of achievement

In study 1 we found a relation between mindset and appraisal of achievement, indicative for the influence of teacher's mindset on how they think about students. The results of our study indicate that overall growth-oriented teachers appreciate increasing marks more than fixed oriented teachers. This is an important finding, given that focusing on increasing achievement has been demonstrated to motivate students (Rheinberg and Engeser, 2010). Focusing on improvement can underscore a student's feelings of competence, which combine with feelings of autonomy and relatedness to form the foundation for intrinsic motivation (Ryan and Deci, 2000). In line with the conclusion of Wilbert and Grúnke (2010), our results indicate the necessity of pointing out improvements. This is especially true for students in the pre-vocational track of secondary education, as they are more likely to be academically challenged and less motivated in school than are students in other tracks. The Dutch system of secondary education has a classification system consisting of several different tracks: pre-vocational education (VMBO), higher general secondary education (HAVO) and pre-university secondary education (VWO). Students in the pre-vocational track exhibit large differences in learning rate, learning style and motivation (Harskamp et al., 2000). Although our results cannot be generalized to all students in this track, they do suggest that, compared to students from the other two tracks, these students tend to be more practical and application-oriented, to prefer educational methods involving concrete rather than theoretical techniques and working forms, and to encounter frequent difficulty in managing their own learning processes (e.g., planning, monitoring, executing/implementing, and evaluating; see Hamstra and Van den Ende, 2006). In combination with self-confidence with regard to ability and motivation, the ability to regulate and control one's own learning process is particularly likely to generate optimal learning outcomes (OECD, 2003). Focussing on growth is therefore of particular importance for students in the pre-vocational track, who generally tend to be less motivated than other students are.

### Feedback

Study 2 focused on the association between mindset and feedback. Feedback has been shown to influence the learning behavior and outcomes of students (Hattie and Timperley, 2007; Lipnevich and Smith, 2009; Geyskens et al., 2012).

Teacher feedback that focuses more on final results (i.e., whether they are sufficient or insufficient) and less on the learning process (i.e., whether there is improvement) might be less effective (Black and Wiliam, 1998; Hattie and Timperley, 2007; Shute, 2008), thus potentially decreasing the results and motivation of students. The literature provides increasing evidence for the importance of such growth-oriented feedback (Mueller and Dweck, 1998; Dweck, 2006; Skipper and Douglas, 2012; Gunderson et al., 2013). Given the frequent occurrence of feedback, their impact is likely to be important, thus underscoring to the importance of providing effective feedback. 26.5% of all feedback interventions were growth-oriented (e.g., pointing out learning questions and hints regarding strategies, possible improvements and alternatives for the choices that students had made) and surprisingly only 2.5% of the oral feedback interventions observed in our study contained comments reflecting personal praise/criticism *on doing (*e.g., “you tried very hard”). Contrary to earlier findings indicating that only half of teachers provide specific feedback (Voerman et al., 2012; Voerman, 2014), all of the teachers in our study (regardless of gender or teaching domain) provided one or more forms of such specific growth-oriented feedback. This discrepancy does not seem to be explained by differences in the definitions used, as Voerman's definition and examples of specific feedback (i.e., “provides information about the learning goal with reference to the task, the processing of the task, or self-regulation, while not being overly elaborate”) largely corresponds to with the concept of growth-oriented feedback. Our overall findings with regard to feedback suggest that teachers use growth feedback to only a limited extent (27.8% of all feedback provided by the teachers was identified as growth feedback).

Given the fact that for example in the educational field formative assessment, closely related to growth feedback, is on the rise (Kneyber and Sluijsmans, 2016), this in an important finding. Formative assessment can only be implemented successfully when teachers are able to provide growth process-oriented feedback.

Contrary to our hypothesis, teachers with a growth mindset generally provided *less* feedback. The results from our sample do not suggest that teachers with a fixed mindset do not guide their students during the process. On the contrary, they provide more feedback overall. Rattan et al. (2012) reported that teachers who believed math-intelligence to be fixed, tended to express their support and encouragement in unproductive ways. They found that those teachers' tended to express both support and encouragement (growth-oriented feedback) in a “comfort-oriented” manner, sending the implicit message: Its' ok- Not everyone can be good at math” (fixed oriented feedback). Contrarily to Rattan, our findings do not suggest that fixed mindset teachers provide support in this comfort-oriented manner. However, in future studies it will be important to explore to which extent such confusing messages occur and to explore the impact of this type of feedback. Not only Rattan, but also Rubie-Davies (2010) reported that teachers might send confusing messages. In her study, some teachers provided messages about positive student characteristics (e.g., trying hard, behaving well, relating well to others) while being negative about their expectations with regard to achievement. This could unintentionally decrease the students' motivation and causing them to have lower expectations regarding their own performance. When teachers report a growth mindset, but do not put their beliefs in action (that is provide growth feedback), students could become demotivated and have lower expectations for their own achievements (Rattan et al., 2012). The teachers in our sample with a growth mindset provided less feedback than those with a fixed mindset. One explanation could be that teachers with a growth mindset are less inclined to urge their students to achieve more or better, being more likely to appreciate their students' efforts as such. Furthermore, self-reported mindset and feedback behavior might be incongruent. This corresponds well to the concept of a “false growth mindset,” in which teachers might claim to have a growth mindset, but do not reflect it in their words or actions (Dweck, 2015).

As indicated by the results from Study 1, teachers with a growth mindset tend to value scores that reflect improvement more highly than teachers with a more fixed mindset. For the growth- oriented teachers, it is not the outcome but the process that is the most important. At the same time, school-based education aims to ensure teaching and learning processes lead to the achievement of certain goals in terms of academic achievement (Rijksoverheid, 1963; Darnon et al., 2012). Therefore the focus of growth-oriented feedback, should include interventions on both process and result.

In our study, we measured the mindset as a general belief, we did not distinguish teacher's mindset in specific situations or toward individual students. Another explanation for the finding that teachers with a more growth-oriented mindset do not provide more growth-oriented feedback, might be that the type of feedback is dependent on both the mindset of the teacher and the characteristics of the individual student. Jager and Denessen (2015) reported that teacher beliefs and their causal attributions toward different low achieving students showed a large within-subject variance. Causal attributions such as attention, effort and interest were described inconsistently for different low-achieving students. Jager and Denessen suggested that attributions are not mere teacher variables but should be studied at the student-specific level.

The relationship between mindset and feedback is even more intricate, however, as students with different mindsets (i.e., growth and fixed) may respond differently to feedback. Compared to the fixed mindset, the growth mindset has been associated with a more effective response to feedback regarding an occasional failure (Dweck, 2007). It might be important to make teachers more explicitly aware of their mindsets, their feedback styles and students' mindsets. If teachers are aware of their own mindsets concerning intelligence, and if they are provided with information on how to provide growth-oriented feedback, this is likely to enhance the effectiveness of their students' learning processes.

### Teacher characteristics

The *gender* of teachers has been shown to be associated with several aspects of their classroom behavior and feedback (Li, 1999; Duffy et al., 2001; Rashidi and Naderi, 2012). The current results indicate that male and female teachers differ in terms of the *appraisal of achievement*, with women tending to value increasing achievements slightly more than men do. However, our findings suggest that female (vs. male) teachers provide a significant smaller proportion of growth-oriented feedback.

Based on the belief that success in some domains (e.g., STEM subjects) depends upon innate ability (Meelissen and Drent, 2009; Michels et al., 2014; Leslie et al., 2015), we predicted that teachers working in such domains would be more oriented toward a fixed mindset. In our study, we found no associations between teaching domain and appraisal of achievements, but contrary to our expectations, we found that STEM teachers provide a higher proportion of growth-oriented feedback then non-STEM teachers. Possibly, the type and amount of feedback needed is domain specific. In the STEM-domain more process feedback might be needed due to the nature of the STEM-domain, regardless of the teachers' (mindset) beliefs. It should be noted that the associations between gender, domain and feedback should be interpreted with caution, as gender and domain overlap in the current sample.

### Limitations

Several limitations of the study should be mentioned. One has to do with the limited sample size in Study 2. However, this sample did not differ from the larger sample regarding the key characteristics appraisal of achievement and mindset on malleability of intelligence. Because of the small sample (*N* = 22) and the number of predictive variables (mindset, gender and domain) it was not feasible to conduct multiple regression analyses.

In addition, our research was conducted on teachers working in one particular track of the Dutch educational system (i.e., pre-vocational secondary education, or VMBO), which includes 55% of all secondary students in the Netherlands (Van Schaik, 2013). Teachers in the pre-vocational track work with students with specific learning characteristics, and they must therefore comply with specific demands with regard to capability, including practice-oriented learning, attention to vocational subjects, integration of several subjects, attention to the learning processes of individual students, a student-oriented approach, customization, and attention to social and emotional development (Van der Rijst et al., 2011). Such demands, especially those having to do with the necessity of attending and customizing instruction to the learning processes of individual students, might be particularly attractive to teachers who believe in growth.

Furthermore, the Theory of Intelligence Questionnaire (Dweck, 2006) is an explicit, self-report measurement. There might therefore be a discrepancy between the responses that teachers entered on this instrument and their actual behavior in the classroom. The participants might have been unwilling or unable to report on their beliefs (Cunningham et al., 2001; Gawronski and De Houwer, 2014), or they might have been biased by a tendency toward socially desirable responses (Hornstra et al., 2010). A different or additional method of measuring mindsets might help to diminish the risk that the results reflect a false growth mindset. Finally, Jager and Denessen (2015) reported that teacher beliefs and causal attributions toward different low achieving students showed a large within-subject variance. A limitation in our study was the absence of mindset of individual students, although results from study 1 suggested that teacher's mindset impacted feedback behavior.

## Conclusions and suggestions for future research

Our results indicate that female teachers and teachers with a more growth-oriented mindset appreciate increasing achievements higher then male teachers or teachers with a more fixed mindset. Our finding highlights the need to pay attention to the mindset of teacher, for example in relation to the increasing use of formative assessment in education. The focus of formative assessment is more on the process than on the result. If formative assessment is on the rise, it is important to be aware that a growth-oriented mindset of the teacher does not necessarily translate into more process-oriented feedback in the classroom. Further investigations are needed to examine the impact of both gender and mindset on appraisal of achievements in the light of the growing importance of process-oriented guidance, assessment and feedback. Our results indicate that teachers use growth feedback in roughly 25% of their feedback interventions. If teachers could be made more explicitly aware of their own mindsets and feedback styles, they might increase the amount of growth feedback that they provide. It would also be helpful for teachers to be aware of the mindsets of their students. The ways in which students react to feedback is likely to depend upon their own mindsets and related emotions. For example, a fixed mindset could lead to maladaptive responses to feedback (Mangels et al., 2006). Future research should include the effect of the mindsets of students on the ways in which they react to feedback and, conversely, how the feedback provided by teachers affects the mindsets of their students.

The overall findings with regard to feedback suggest that although all teachers in our sample provided growth-oriented feedback, they did so only to a limited extent. Furthermore, we showed a negative correlation between mindset (through a self-reported questionnaire) and the amount of feedback: A more growth-oriented mindset is no guarantee for more growth-oriented feedback. These are important findings for teaching practice, given the impact of feedback on the learning outcomes and motivation of students. Although motivation was not the focus of this study, it is closely related to feedback (Mueller and Dweck, 1998). Future investigations are needed in order to examine the effects of several types of feedback on motivation and emotions. Additional investigation in other educational tracks is needed in order to broaden the existing knowledge concerning the mindsets of teachers and their use of feedback, in addition to examining whether the current results are specific to the pre-vocational track or whether they can be generalized to all teachers.

## Ethics statement

All of the procedures of the study were in strict compliance with the ethical guidelines of the faculty of the VU university. Participants were asked to provide informed consent before taking part. In schools there was a video-protocol: when their children enter secondary school, parents are asked to provide written consent for video/audio recording. If they did not provide this written consent, the students were excluded from video-taping.

## Author contributions

EdKP, FvW, and LK conceptualized the studies, EdKP acquired the data, all authors contributed to the data analysis approach, EdKP and FvW performed the data analysis, EdKP wrote the manuscript with contributions from all other authors.

### Conflict of interest statement

The authors declare that the research was conducted in the absence of any commercial or financial relationships that could be construed as a potential conflict of interest.
