# Supplementary material for: Teacher Mindsets Concerning the Malleability of Intelligence and the Appraisal of Achievement in the Context of Feedback
Source: Front Psychol. 2017 Sep 21;8:1594. doi: 10.3389/fpsyg.2017.01594 (PMC5613779; doi:10.3389/fpsyg.2017.01594)
Supplement: Supplementary file 1 [file Appendix1.pdf]

APPENDIX 1

Student	*mark on Test 1*	*Test 2*	*Test 3*	*Teacher's Appraisal of Test 3* (--, -, +/-, +, ++)
**1**	8.5	8.0	7.5	→ ☐
**2**	4.0	4.5	5.0	→ ☐
**3**	2.5	2.5	2.5	→ ☐
**4**	6.0	5.5	5.0	→ ☐
**5**	5.0	5.0	5.0	→ ☐
**6**	7.5	7.5	7.5	→ ☐
**7**	3.5	3.0	2.5	→ ☐
**8**	6.5	7.0	7.5	→ ☐
**9**	2.5	4.0	5.0	→ ☐
**10**	9.0	5.0	7.0	→ ☐
**11**	3.0	5.5	6.5	→ ☐
**12**	9.0	9.0	6.5	→ ☐

Figure 1. Adapted Student Evaluation Task (based on Rheinberg, 1980)

1-8 are the original marks. The set '1,5-2,0-2,5' was used in the original tests by Rheinberg, but not in this study. In Rheinberg's *study: Increasing marks: Items 2, 8; Stagnating: Items 3,5,6; Decreasing: Items 1,4,7.* In our study, Items *9-12 are added*: Increasing marks: Items 2,8,9,11; Non-increasing, sufficient marks: Items 1,6,10,12; Non-increasing insufficient marks: Items 3,4,5,7.
