# Supplementary material for: Teacher Mindsets Concerning the Malleability of Intelligence and the Appraisal of Achievement in the Context of Feedback
Source: Front Psychol. 2017 Sep 21;8:1594. doi: 10.3389/fpsyg.2017.01594 (PMC5613779; doi:10.3389/fpsyg.2017.01594)
Supplement: Supplementary file 2 [file Appendix2.pdf]

APPENDIX 2

Table 1. *Teaching domain and total length of the video fragment for each individual teacher (n=23)*

Teacher ID	*Teaching domain (mathematics or Dutch)*	*Total length of video fragment (in minutes)*
1	mathematics	33
2	Dutch	20
3	mathematics	36
4	mathematics	29
5	Dutch	24
6	Dutch	37
7	mathematics	41
8	mathematics	26
9	Dutch	30
10	mathematics	37
11	mathematics	37
12	Dutch	40
13	mathematics	36
14	Dutch	29
15	Dutch	38
16	Dutch	44
17	Dutch	40
18	mathematics	42
19	Dutch	38
20	mathematics	0
21	mathematics	35
22	mathematics	36
23	Dutch	31

**ID code**						
	**Fragment**					
**Fixed praise/criticism personal comments on being**						
**Growth-oriented praise/criticism personal comments on doing**						
**Fixed feedback on *what* results were achieved**						
Confirming what is right so far	Without additional information					
Idem	With additional information					
Saying what is missing/wrong	Without additional information					
Idem	With additional information					
Giving the correct solution/answer	Without additional information					
Idem	With additional information					
Mentioning the criteria to be met						
**Growth-oriented feedback on *how* results were achieved**						
Encouraging students to analyse or describe the problem more precisely						
Asking questions about what the student has already done						
Asking open questions to stimulate the process of thinking						
Clarifying or simplifying the problem by dividing it into small steps						
Giving hints, cues, examples						
Making suggestions for improvement						
Making suggestions to monitor the process of learning						
**Other**process						
Asking questions like, ‘Do you understand?’, ‘Is everything clear?’						
Asking questions for specific knowledge						

Figure 2. Adapted Teacher Feedback Behaviour scoring form (based on Sol & Stokking, 2008, UU)
